# Convalescent troponin and cardiovascular death following acute coronary syndrome

**DOI:** 10.1136/heartjnl-2019-315084

**Published:** 2019-07-23

**Authors:** Philip D Adamson, David McAllister, Anna Pilbrow, John William Pickering, Katrina Poppe, Anoop Shah, Gillian Whalley, Chris Ellis, Nicholas L Mills, David E Newby, Chris Pemberton, Richard W Troughton, Rob N Doughty, A Mark Richards

**Affiliations:** 1 Christchurch Heart Institute, University of Otago Christchurch, Christchurch, New Zealand; 2 British Heart Foundation Centre for Cardiovascular Science, University of Edinburgh, Edinburgh, UK; 3 McAllister, David, Edinburgh, UK; 4 Medicine, University of Otago, Christchurch, New Zealand; 5 Epidemiology & Biostatistics, University of Auckland, Auckland, New Zealand; 6 BHF/University Centre for Cardiovascular Science, Royal Infirmary of Edinburgh, Edinburgh, UK; 7 Department of Medicine, University of Otago, Dunedin, New Zealand; 8 Cardiology, Greenlane CVS Services, Auckland City Hospital, Auckland, New Zealand; 9 BHF Centre for Cardiovascular Sciences, The University of Edinburgh, Edinburgh, UK; 10 Centre for Cardiovascular Sciences, University of Edinburgh, Edinburgh, UK; 11 Cardiology, Christchurch Hospital, Christchurch, New Zealand; 12 Medicine, University of Otago, Christchurch, New Zealand; 13 Department of Medicine, University of Auckland, Auckland, New Zealand

**Keywords:** acute coronary syndromes

## Abstract

**Objectives:**

High-sensitivity cardiac troponin testing is used in the diagnosis of acute coronary syndromes but its role during convalescence is unknown. We investigated the long-term prognostic significance of serial convalescent high-sensitivity cardiac troponin concentrations following acute coronary syndrome.

**Methods:**

In a prospective multicentre observational cohort study of 2140 patients with acute coronary syndrome, cardiac troponin I concentrations were measured in 1776 patients at 4 and 12 months following the index event. Patients were stratified into three groups according to the troponin concentration at 4 months using the 99th centile (women>16 ng/L, men>34 ng/L) and median concentration of those within the reference range. The primary outcome was cardiovascular death.

**Results:**

Troponin concentrations at 4 months were measurable in 99.0% (1759/1776) of patients (67±12 years, 72% male), and were ≤5 ng/L (median) and >99th centile in 44.8% (795) and 9.3% (166), respectively. There were 202 (11.4%) cardiovascular deaths after a median of 4.8 years. After adjusting for the Global Registry of Acute Coronary Events score, troponin remained an independent predictor of cardiovascular death (HR 1.4, 95% CI 1.3 to 1.5 per doubling) with the highest risk observed in those with increasing concentrations at 12 months. Patients with 4-month troponin concentrations >99th centile were at increased risk of cardiovascular death compared with those ≤5 ng/L (29.5% (49/166) vs 4.3% (34/795); adjusted HR 4.9, 95% CI 3.8 to 23.7).

**Conclusions:**

Convalescent cardiac troponin concentrations predict long-term cardiovascular death following acute coronary syndrome. Recognising this risk by monitoring troponin may improve targeting of therapeutic interventions.

**Trial registration number:**

ACTRN12605000431628;Results.

## Introduction

In the assessment of patients with suspected acute coronary syndrome, international guidelines recommend serial measurement of cardiac troponin in accordance with the universal definition of myocardial infarction.^1^ In this acute context, increased troponin concentrations above the 99th centile reference limit have consistently demonstrated a correlation between infarct size and subsequent prognosis.^2^ Recently approved high-sensitivity troponin assays have also revealed a gradient of risk associated with concentrations below the diagnostic threshold at the time of initial presentation.^3–5^ However, no guidance is offered on measuring troponin once a diagnosis is established, and long-term risk stratification following hospital discharge is largely dependent on left ventricular function and the presence of residual inducible ischaemia.^6^

Despite the widely accepted view that troponin concentrations typically return to baseline levels within 2 weeks, the exact time course of resolution of cardiac troponin following myocardial infarction remains unclear and several reports have described persistent elevations up to 7 weeks following initial presentation.^7 8^ Plausible contributory mechanisms include delayed healing of the ruptured plaque, persistent left ventricular dysfunction or asymptomatic ischaemia.^8 9^

There is now emerging evidence from posthoc analysis of clinical trial populations that increased troponin concentrations during the early and intermediate postmyocardial infarction period confer greater risk of recurrent cardiovascular events.^10 11^ However, the time course and clinical implications of persistent myocardial injury following acute coronary syndrome remain uncertain. We sought to determine the long-term prognostic significance of serial convalescent high-sensitivity cardiac troponin I concentrations in a large prospective study incorporating detailed clinical characterisation following acute coronary syndrome.

## Methods

### Study population

From 2002 to 2009, 2140 patients were enrolled into the Coronary Disease Cohort Study (CDCS), a prospective, multicentre (Christchurch and Auckland City Hospitals) observational cohort study in New Zealand designed to explore the determinants of long-term risk following acute coronary syndrome.^12^ Inclusion required hospitalisation with an acute coronary syndrome diagnosed according to previously reported criteria (online supplementary appendix).^13^ The CDCS conforms to the principles outlined in the Declaration of Helsinki and was approved by the New Zealand Multi-Region Ethics Committee. All participants provided written, informed consent.

10.1136/heartjnl-2019-315084.supp1 Supplementary data

### Clinical assessments and outcomes

Patients underwent comprehensive clinical assessment including a 12-lead ECG, echocardiography and blood sampling at 1, 4 and 12 months following the index event, with additional annual review for at least 2 years (online supplementary table S1). Echocardiographic assessment was performed according to the recommendations of the American Society of Echocardiography.^14^ Long-term clinical events (hospitalisations and mortality) were determined from the New Zealand National Health Information Services databases, comprising comprehensive population-wide records linked by a unique identifier. All deaths were adjudicated for cause by a cardiologist blind to study troponin measurements. Further details regarding endpoint classification are provided in the online supplementary appendix.

### Biomarker analysis

**Figure 1:**
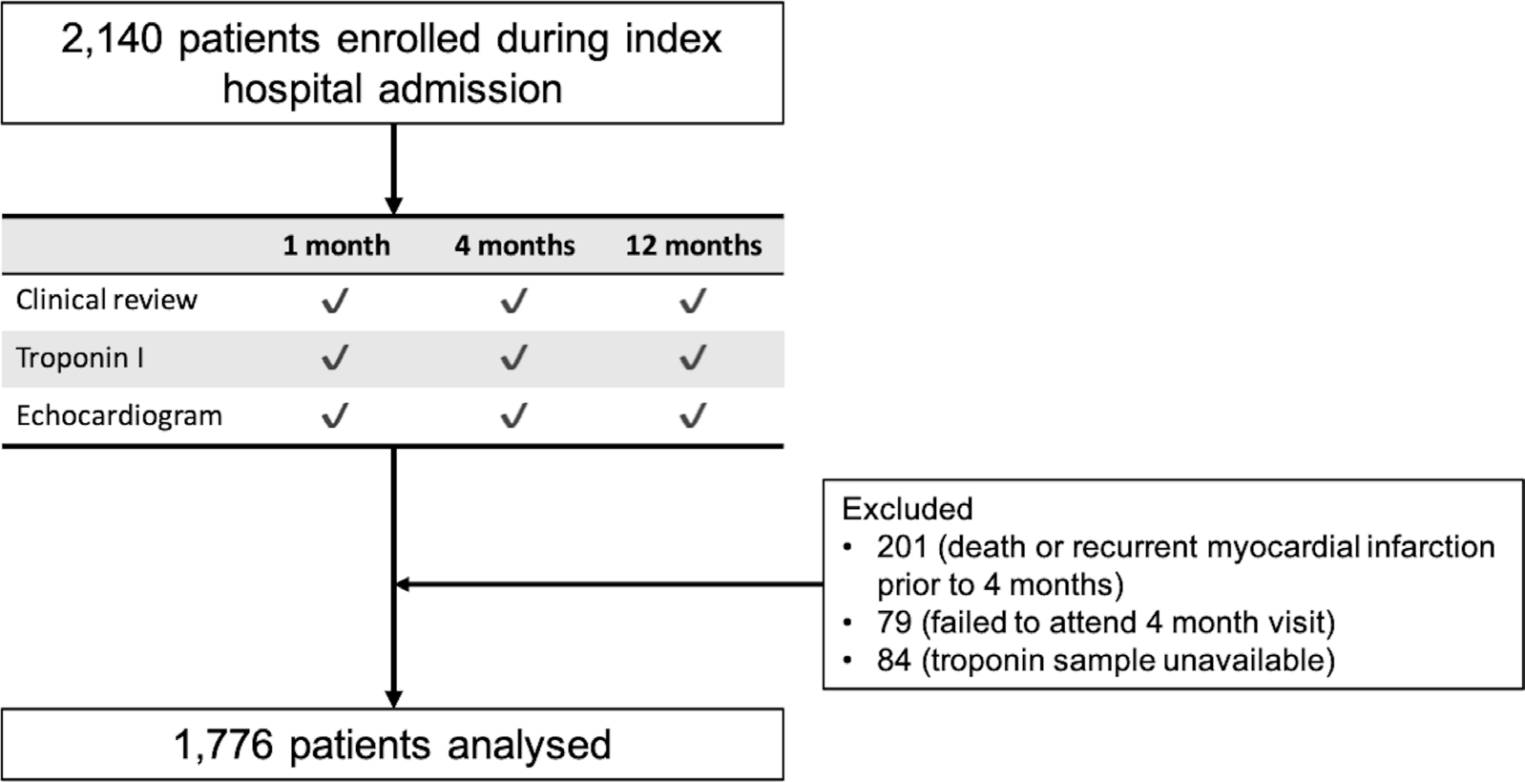
Study profile.

Following collection and centrifugation, EDTA-plasma blood samples were stored at −80°C until analysed. High-sensitivity cardiac troponin I concentrations were determined using the ARCHITECT*_STAT_* assay (Abbott Laboratories, Abbott Park, Illinois, USA), which has a limit of detection of 1.2 ng/L, coefficient of variation <10% at 3.0 ng/L and 99th centile upper reference limit of 16 ng/L for women and 34 ng/L for men.^15 16^ To allow for variation in hospital stay and ensure resolution of the initial ischaemic event, the convalescent troponin concentration was defined as that obtained at the second study visit at 4 months. For the purposes of this analysis, patients were excluded if they had experienced a recurrent myocardial infarction prior to this visit (n=201) or failed to attend (n=79) or have a troponin measurement performed (n=84) at the 4-month visit (figure 1).

### Clinical endpoints

The primary endpoint was cardiovascular death following the 4-month visit. Secondary endpoints included all-cause death, non-cardiovascular death, fatal or non-fatal myocardial infarction, heart failure hospitalisation, the composite of cardiovascular death or non-fatal myocardial infarction and hospitalisation with non-fatal stroke.

### Statistical analysis

Patient characteristics were reported by groups determined from the 4-month troponin concentration rounded to the nearest integer value. Groups 1 and 2 comprised those below and above the median troponin concentration excluding those in group 3 with concentrations above the sex-specific 99th centiles. Cox regression results are reported as HRs per doubling of troponin concentration, both unadjusted and following adjustment for the Global Registry of Acute Coronary Events (GRACE) risk score at time of discharge following index hospitalisation.^17^

In determining the relationship between troponin concentrations and treatment, we defined optimal medical therapy at discharge as per current clinical guidelines.^18^

Clinical determinants of cardiac troponin concentrations were determined from linear mixed models. The slope (time since initial presentation) and intercept were included as random effects. Time varying covariates were centred within-person as per Curran and Bauer.^19^ In a sensitivity analysis, we repeated the modelling with time as a categorical variable, using an unstructured correlation matrix for the within-person errors.

The negative predictive values for the primary outcome were established across a range of troponin concentrations starting at 2 ng/L with a negative predictive value at 5 years of 97.5% evaluated as an acceptable threshold indicator of low risk.^20^

In a landmark analysis following the 12-month visit, the additional prognostic information provided by serial changes in troponin concentration was determined by including 12-month troponin concentrations in a Cox proportional hazards model adjusting for 4-month troponin concentrations. Patients were included in this analysis if they had troponin results available from all three study visits and had not experienced a recurrent myocardial infarction prior to the 12-month visit (n=1513).

Data are presented as mean (±SD) or median (IQR) with 95% CI with two-tailed p values reported. Statistical analysis was performed using R V.3.4.3 (R Foundation for Statistical Computing, Vienna, Austria). Additional details of the statistical analyses undertaken are described in the online supplementary appendix.

## Results

After excluding patients with recurrent myocardial infarction prior to the 4-month visit, 1776 patients (66.4±12.0 years, 72% male) had troponin concentrations measured at the second study visit (128 (IQR 116–140) days) and were included in the analysis (table 1). The average duration of follow-up after the 4-month visit was 4.8 years with a maximum follow-up of 9.2 years. Patients had a substantial burden of cardiovascular risk factors including hypertension (51%), hypercholesterolaemia (55%) and diabetes mellitus (16%). The average estimated risk of death at 6 months as determined from the discharge GRACE score^19^ was 5.2%±0.8%.

**Table 1:** Baseline characteristics of patients with acute coronary syndrome stratified by cardiac troponin I concentration at 4 months

	Total CDCS	Biomarker cohort	≤5 ng/L	6 ng/L–99th centile	>99th centile
n	2140	1776	795	815	166
Age, years	66.5 (12.3)	66.4 (12.0)	63.7 (11.4)	68.2 (11.9)	70.8 (12.9)
Sex, male	1531 (71.5)	1282 (72.2)	534 (67.2)	661 (81.1)	87 (52.4)
Past medical history*					
Hypertension	1104 (52.0)	905 (51.4)	365 (46.4)	432 (53.5)	108 (65.1)
Hypercholesterolaemia	1135 (54.3)	952 (54.5)	440 (56.1)	426 (53.2)	86 (53.1)
Diabetes mellitus	353 (16.5)	276 (15.6)	96 (12.1)	142 (17.4)	38 (22.9)
Smoking status					
Never smoked	787 (36.8)	669 (37.7)	290 (36.5)	316 (38.8)	63 (38.0)
Ex-smoker	1218 (56.9)	999 (56.2)	448 (56.4)	465 (57.1)	86 (51.8)
Current smoker	135 (6.3)	108 (6.1)	57 (7.2)	34 (4.2)	17 (10.2)
Prior myocardial infarction	635 (29.9)	504 (28.5)	168 (21.2)	268 (33.0)	68 (41.5)
Prior stroke	257 (12.1)	200 (11.3)	66 (8.3)	103 (12.7)	31 (18.7)
Prior congestive heart failure	201 (9.4)	154 (8.7)	27 (3.4)	95 (11.7)	32 (19.5)
Status at presentation					
Heart rate, bpm	73.1 (20.3)	72.7 (20.6)	70.7 (19.3)	73.0 (20.8)	81.2 (23.2)
Systolic blood pressure, mm Hg	134.9 (20.3)	135.3 (20.6)	135.1 (20.0)	133.4 (21.2)	148.3 (20.3)
Killip class					
I	1577 (73.8)	1329 (75.0)	664 (83.7)	569 (69.9)	96 (57.8)
II	526 (24.6)	419 (23.6)	124 (15.6)	233 (28.6)	62 (37.3)
III/IV	33 (1.5)	25 (1.4)	5 (0.6)	12 (1.5)	8 (4.8)
eGFR, mL/min	65.6 (19.9)	66.1 (19.5)	71.5 (16.9)	62.9 (20.1)	55.5 (20.5)
ST depression	471 (22.1)	386 (21.9)	112 (14.1)	218 (26.9)	56 (33.9)
Positive cardiac biomarker (index admission)	1740 (81.4)	1422 (80.1)	575 (72.3)	691 (84.9)	156 (94.0)
No. of diseased vessels†					
0	105 (6.4)	90 (6.5)	63 (9.4)	21 (3.4)	6 (5.8)
1	450 (27.5)	387 (27.8)	206 (30.7)	155 (25.0)	26 (25.2)
2	487 (29.7)	413 (29.6)	194 (29.0)	187 (30.2)	32 (31.1)
3	596 (36.4)	503 (36.1)	207 (30.9)	257 (41.5)	39 (37.9)
In-hospital percutaneous coronary intervention	1080 (50.5)	932 (52.5)	442 (55.6)	427 (52.4)	63 (38.0)
LVEF at 4 months, %		59.0 (52.0, 65.0)	61.0 (56.0, 66.0)	58.0 (49.0, 64.0)	51.0 (40.0, 61.0)
Medications at discharge					
Aspirin	2087 (97.6)	1736 (97.9)	781 (98.4)	798 (98.0)	157 (94.6)
Clopidogrel	1244 (58.2)	1053 (59.4)	489 (61.6)	479 (58.8)	85 (51.2)
Renin-angiotensin system-blocking agents‡	1334 (62.4)	1095 (61.7)	419 (52.8)	553 (67.9)	123 (74.1)
Beta-blocker	1856 (86.7)	1554 (87.5)	706 (88.8)	710 (87.1)	138 (83.1)
Statin	1948 (91.1)	1623 (91.5)	743 (93.6)	739 (90.8)	141 (84.9)
Optimal medical therapy at discharge¶		884 (49.8)	411 (51.7)	406 (49.8)	67 (40.4)
Index diagnosis					
Unstable angina	567 (26.5)	495 (27.9)	294 (37.0)	177 (21.7)	24 (14.5)
NSTEMI	1089 (50.9)	864 (48.6)	377 (47.4)	388 (47.6)	99 (59.6)
STEMI	484 (22.6)	417 (23.5)	124 (15.6)	250 (30.7)	43 (25.9)
Time from index admission to 4-month visit, days	128.0 (116.0, 140.0)	128.0 (116.0, 140.0)	129.0 (117.0, 142.0)	126.0 (115.0, 138.0)	128.0 (117.0, 141.8)
GRACE score	111.0 (31.7)	109.9 (30.8)	99.4 (25.2)	116.2 (30.9)	129.9 (36.2)
GRACE score tertile¶					
Low	614 (30.6)	517 (31.0)	286 (38.1)	204 (26.6)	27 (17.8)
Intermediate	675 (33.7)	588 (35.2)	311 (41.5)	240 (31.2)	37 (24.3)
High	716 (35.7)	565 (33.8)	153 (20.4)	324 (42.2)	88 (57.9)

*See https://www.outcomes-umassmed.org/grace/grace_risk_table.aspx).

†Determined at time of index hospital admission.

‡Determines from diagnostic coronary angiography performed during index admission.

§Including ACE inhibitors or angiotensin receptor blockers.

¶GRACE score tertiles determined according to index diagnosis: NSTEACS, low<88, intermediate 89–118, high≥119; STEMI, low<99, intermediate 100–127, high≥128.

§Optimal medical therapy defined as a prescription for all of aspirin, clopidogrel and a statin. In addition, prescription of a beta-blocker was required if the LVEF was ≤40%. An ACE inhibitor or angiotensin receptor blocker was also required if the LVEF was ≤40% or the patient had a diagnosis of hypertension or diabetes mellitus.

CDCS, Coronary Disease Cohort Study; eGFR, estimated glomerular filtration rate; GRACE, Global Registry of Acute Coronary Events; LVEF, left ventricular ejection fraction; NSTEMI, non-ST-segment elevation myocardial infarction; STEMI, ST-segment elevation myocardial infarction.

### Distribution and trends in convalescent troponin concentrations

**Figure 2:**
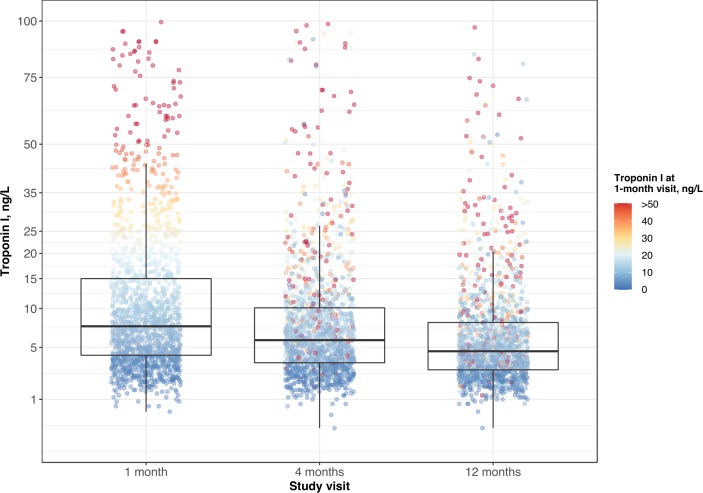
Trends in troponin concentration over 1 year following acute coronary syndrome. Patients with samples available at all time-points and without recurrent myocardial infarction prior to the 12-month visit were included in this analysis (n=1513). Each individual is represented as a circle coloured according to their troponin concentration at the 1-month study visit. This demonstrates both average and individual change in troponin concentrations over time. The whiskers of the boxplots extend to the highest or lowest values not greater than 1.5 times the IQR of troponin concentrations.

Median troponin concentrations declined at each successive visit over the first year with median values of 8.2 (IQR 4.2–17.8) ng/L, 6.1 (IQR 3.6–11.5) ng/L and 4.8 (IQR 2.9–8.6) ng/L at the 1-month, 4-month and 12-month visits, respectively (p<0.0001; figure 2). The proportion of patients with troponin concentrations above the sex-specific 99th centile was 16.0% (1-month visit), 9.3% (4-month visit) and 5.9% (12-month visit).

At the 4-month visit, plasma troponin I concentrations were above the limit of detection and the sex-specific 99th centile threshold in 1759 (99.0%) and 166 (9.3%) patients, respectively (online supplementary figure S1). The median troponin concentration of those within the reference range at the 4-month visit was 5.5 (women 4.0, men 6.0) ng/L. The extent of myocardial injury during the index admission (as determined by the peak creatine kinase concentration) was only weakly associated with troponin concentration at 4 months (r=0.19, p<0.0001). In contrast, troponin measured at 4 and 12 months were much more strongly correlated (r=0.78, p<0.0001; online supplementary figure S2). After adjustment for age, sex and left ventricular ejection fraction (LVEF), 4-month troponin was not associated with a change in prescription of optimal medical therapy (OR 0.99 per doubling of troponin, 95% CI 0.92 to 1.08, p=0.8741), with 49.7% receiving optimal medical therapy.

### Convalescent troponin and cardiovascular outcomes

**Figure 3:**
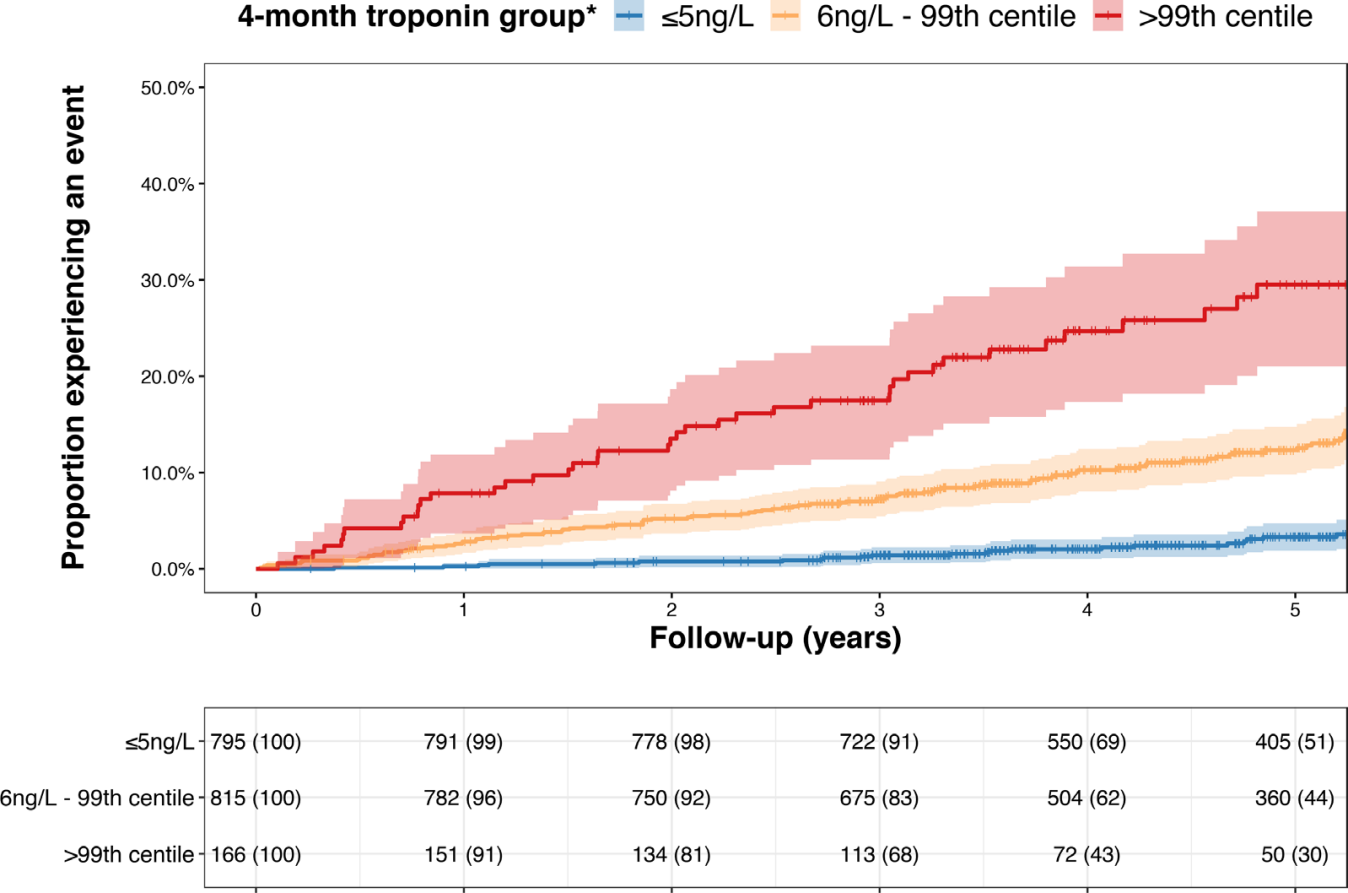
Cumulative incidence of cardiovascular death according to 4-month troponin concentration Cumulative event curves for cardiovascular death according to troponin concentrations determined at the 4-month visit. Each cross-hair indicates when a subject is censored from further follow-up. The number at risk (% in group remaining) for each yearly interval is given for each troponin group. Follow-up begins from date of 4-month visit. *For descriptive purposes, troponin concentrations have been rounded to nearest integer value. Therefore ≤5 ng/L includes all patients <5.5 ng/L.

During follow-up, there were 375 deaths of which 202 were secondary to cardiovascular causes. A threshold of ≤5 ng/L applied at 4 months identified 45% of the study cohort (53% of women, 42% of men) with a negative predictive value for cardiovascular death at 5 years of 97.1% (95% CI 95.5 to 98.6; online supplementary figure S3) among both women and men. The negative predictive value decreased at higher troponin concentrations and was <97% at concentrations of ≥6 ng/L. In the prediction of this endpoint, the c-statistic for convalescent troponin (0.745, 95% CI 0.707 to 0.745) was greater than that provided by knowledge of the extent of myocardial injury during the index event as determined from peak concentrations during the index admission of either creatine kinase (c-statistic 0.562, 95% CI 0.513 to 0.562), troponin I (c-statistic 0.585, 95% CI 0.519 to 0.585) or troponin T (c-statistic 0.557, 95% I 0.504 to 0.557; p<0.001 for all comparisons with 4 month troponin, online supplementary figure S4). This was also greater than the predictive value of LVEF as determined at the 4-month visit (c-statistic 0.679, 95% CI 0.621 to 0.738, p=0.0094). In unadjusted analysis, 4-month troponin concentrations demonstrated a robust association with cardiovascular death (HR 1.50, 95% CI 1.42 to 1.59 per doubling of troponin; p<0.0001, online supplementary figure S5). This association remained after adjusting for the discharge GRACE score (HR 1.36, 95% CI 1.26 to 1.47 per doubling; p<0.0001) or an extended risk model incorporating additional clinical, biochemical and echocardiographic factors during follow-up to 4 months (HR 1.35, 95% CI 1.15 to 1.58; p=0.0002, online supplementary table S2). After adjustment for the discharge GRACE score, patients with convalescent troponin concentrations above the 99th centile had a fivefold increased risk of cardiovascular death compared with those ≤5 ng/L (HR 4.87; 95% CI 2.96 to 7.99, p<0.0001, figure 3). The association between troponin and secondary endpoints was greatest for cardiac events but remained for all-cause death and non-cardiovascular death (table 2).

**Table 2:** Cox proportional hazards for secondary endpoints according to troponin adjusted for GRACE discharge risk

	≤5 ng/L	6 ng/L–99th centile		>99th centile*	
	(n=795)	(n=815)	HR (95% CI)	(n=166)	HR (95% CI)
Cardiovascular death	34 (4.3)	119 (14.6)	2.52 (1.65 to 3.85)	49 (29.5)	4.87 (2.96 to 7.99)
Cardiac death	25 (3.1)	92 (11.3)	2.51 (1.54 to 4.07)	43 (25.9)	5.50 (3.17 to 9.57)
All-cause death	81 (10.2)	216 (26.5)	1.90 (1.44 to 2.51)	78 (47.0)	3.34 (2.36 to 4.73)
Cardiovascular death or non-fatal myocardial infarction	123 (15.5)	236 (29.0)	1.58 (1.25 to 2.01)	74 (44.6)	2.51 (1.82 to 3.44)
Non-cardiovascular death	47 (5.9)	97 (11.9)	1.48 (1.02 to 2.16)	29 (17.5)	2.30 (1.38 to 3.81)
Fatal or non-fatal myocardial infarction	102 (12.8)	168 (20.6)	1.38 (1.06 to 1.80)	53 (31.9)	2.23 (1.55 to 3.21)
Heart failure hospitalisation	53 (6.7)	165 (20.2)	2.09 (1.48 to 2.95)	66 (39.8)	3.98 (2.62 to 6.05)
Non-fatal stroke	45 (5.7)	68 (8.3)	1.12 (0.73 to 1.71)	13 (7.8)	0.98 (0.48 to 2.02)

*99th centile determined using sex-specific cut points (women>16 ng/L, men>34 ng/L).

Values are n (%) unless otherwise stated.

GRACE, Global Registry of Acute Coronary Events.

### Serial measurement of troponin and cardiovascular outcomes

**Figure 4:**
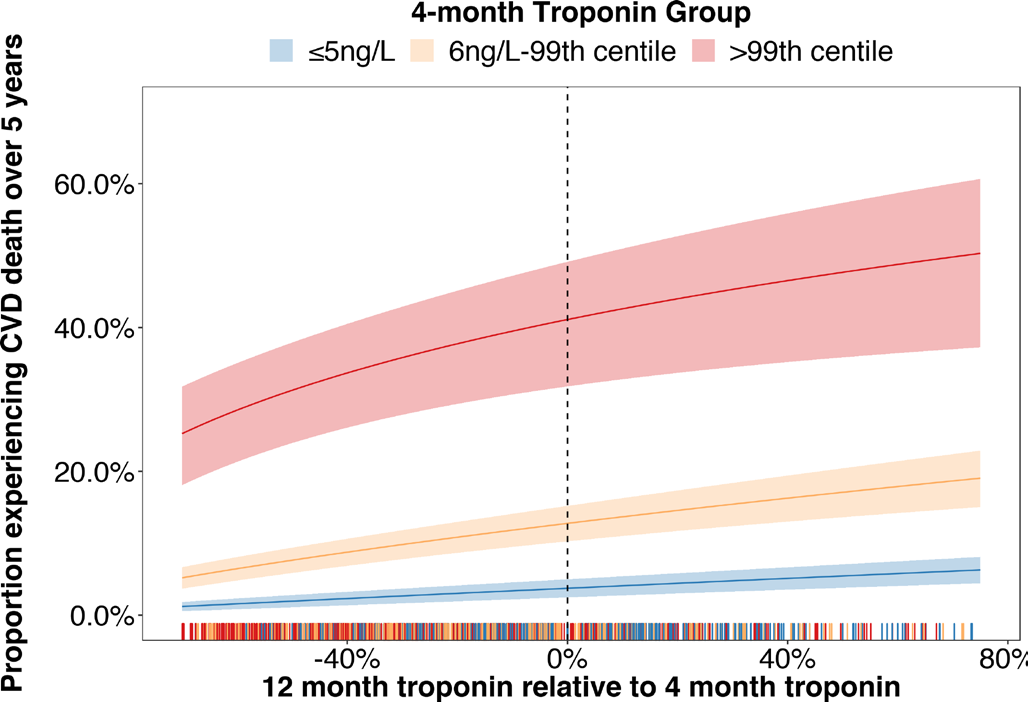
Risk of cardiovascular death at 5 years according to 4-month troponin concentration and relative change in troponin concentration to 12 months. The estimated 5-year risk of cardiovascular death in relation to interval change in troponin concentrations is presented and grouped according to 4-month troponin concentrations (≤5 ng/L—blue, 6 ng/L–99^th^ centile—orange, >99^th^ centile—red). The 95% CIs for these estimates are demonstrated within the correspondingly shaded regions. The rug-plot shown along the x-axis depicts each occurrence of the specific combination of 4-month troponin and relative change to 12 months within the study cohort. For descriptive purposes, troponin concentrations have been rounded to nearest integer value. Therefore, ≤5 ng/L includes all patients <5.5 ng/L. Additional details regarding this statistical model are provided in the online supplementary appendix. CVD, cardiovascular disease.

In a landmark analysis restricted to events following the 12-month visit, troponin concentrations at the 12-month visit provided additional information regarding the risk of cardiovascular death over and above the 4-month troponin concentration (p-interaction=0.0200; online supplementary table S3). The majority of patients (46.9%) demonstrated a ≥20% decline in troponin concentrations during this time period while in 16.1% of cases the troponin concentration increased by at least 20%. Increasing or decreasing troponin concentrations from 4 to 12 months were associated with rising or falling risks of cardiovascular death in all three groups although the absolute 5-year risk remained <10% in those ≤5 ng/L and >25% in those >99th centile at the 4-month visit (figure 4).

### Clinical determinants of cardiac troponin in convalescence

Older patients had higher troponin concentrations at 4 months (1.25-fold difference per SD in age; 95% CI 1.19 to 1.30) and experienced a slower decline in troponin (1.18-fold difference per SD in age; 95% CI 1.13 to 1.23, online supplementary figure S6). While men had higher troponin concentrations than women (6.6 (IQR 3.8–12.0) ng/L vs 5.1 (IQR 3.0–9.7) ng/L; p<0.0001), the rate of decline was similar in both sexes. In age, sex and diagnosis-adjusted models, troponin was higher in patients with lower LVEF (0.74-fold difference per SD in ejection fraction; 95% CI 0.71 to 0.77) and higher heart rates (1.08-fold difference per SD in heart rate; 95% CI 1.02 to 1.13). Within-patient decrease in LVEF and increase in systolic blood pressure and heart rate were also associated with increase in troponin from 4 to 12 months (online supplementary table S4).

## Discussion

In the evaluation of patients following hospitalisation with acute coronary syndrome, convalescent high-sensitivity cardiac troponin conveys powerful long-term prognostic information that adds substantially to currently recognised clinical risk predictors and scores. Indeed, even after adjustment for the GRACE score, we identified a fivefold increase in risk of cardiovascular death, associated with increasing convalescent troponin concentrations. This gradient of risk was continuous across the range of troponin concentrations and was greatest for cardiac events including cardiac death and heart failure hospitalisation.

Our study has several important strengths. First, the availability of comprehensive outcome data with more than 200 cardiovascular deaths during up to 9 years of follow-up is a major asset and enabled us to define the long-term prognostic significance of convalescent measurement of troponin. Second, the deliberately broad inclusion criteria ensured recruitment of a diverse and representative cohort of real-world patients with acute coronary syndrome. The even distribution of patients across low-risk, intermediate-risk and high-risk groups as determined by the GRACE risk score offers strong evidence as to the generalisability of our findings. Third, detailed characterisation with repeated clinical, biochemical and echocardiographic assessments at three time points allowed us to explore patterns of change in convalescent troponin concentrations, and how these correlate with both patient characteristics and long-term outcomes.

Our findings have clear potential to improve follow-up and management of patients discharged following acute coronary syndrome. We have shown that a single measurement of cardiac troponin during convalescence from an acute coronary syndrome can identify nearly half of patients as low-risk, with 0.5% annual incidence of cardiovascular death. A further 1 in 10 patients have persistent myocardial injury and are identified as very high risk, with annual event rates approaching 10%, for whom careful assessment and more intensive treatment may be warranted. Furthermore, we have demonstrated that for those at intermediate risk, repeated measurement of cardiac troponin concentrations at 12 months can be used to improve risk estimation and enable prognostic reclassification into either low-risk or high-risk groups.

Convalescent troponin concentrations are not simply a surrogate for the extent of myocardial injury sustained during the index coronary event as evidenced by the weak correlation between peak troponin during the index hospitalisation and 4-month troponin. Neither are they simply a reflection of left ventricular function. Instead, they provide greater prognostic information than either of these traditional risk indicators and are additive to all other clinical, biochemical and echocardiographic risk factors that might typically be available to clinicians when making treatment decisions. Furthermore, within this real-world observational study, we have revealed a substantial treatment gap whereby less than half of patients with troponin concentrations >99th centile received all guideline-indicated medications. This gap likely reflects an underappreciation of risk by both clinicians and patients and signifies a clear clinical rationale for the prognostic information troponin offers.

Although our findings are the first from a prospective observational cohort study, they are consistent with posthoc analyses of randomised controlled trials of both stable coronary disease^21 22^ and recent myocardial infarction.^10 11^ These latter two studies include the Pravastatin or Atorvastatin Evaluation and Infection Therapy–Thrombolysis in Myocardial Infarction 22 (PROVE-IT TIMI 22) trial of pravastatin following acute coronary syndrome, and the Examination of Cardiovascular Outcomes with Alogliptin versus Standard of Care (EXAMINE) trial, testing the cardiovascular safety of alogliptin in patients with type 2 diabetes mellitus. The prevalence of troponin concentrations >99th centile was 22.2% at 4 months in PROVE-IT TIMI 22% and 11.7% at 6 months in EXAMINE. Despite fewer overall events and shorter durations of follow-up, both studies identified strong associations between increasing troponin concentrations and cardiovascular risk. Interestingly, within the former study, troponin also appeared to identify those who derived greatest benefit from pravastatin, with an absolute risk reduction of 3.5% (95% CI 0.41 to 6.59) in the subset with high troponin concentrations compared with 0.4% (95% CI −1.0% to 0.9%) in the remainder. The use of troponin as a potentially useful target of therapeutic intervention has also previously been described in high-risk patients without established coronary heart disease treated with statin therapy^23^ and the sodium glucose cotransporter 2 inhibitor canagliflozin,^24^ where the reduction in troponin concentration on treatment was associated with the magnitude of cardiovascular benefit.

Taken alongside these reports, our study has important clinical implications regarding the measurement of troponin following an acute coronary syndrome. The American Heart Association estimates that 720 000 individuals within the USA alone will experience a first fatal or non-fatal myocardial infarction during 2018.^25^ A further 335 000 will have a recurrent event.^25^ This sizeable residual risk persists despite major therapeutic advances including potent preventative therapies and early revascularisation. Improving long-term outcomes in a cost-effective manner will require improved therapeutic targeting. Within this context, troponin testing is inexpensive and already widely available in clinical practice. In the face of resource constraints, troponin concentrations could be used to guide the use of more costly investigations and in targeting treatments at the subgroup of patients identified to be at greatest risk of adverse events. We have shown that troponin concentrations increase as LVEF declines, and identifying this pattern could prompt clinicians to reassess cardiac function and encourage uptitration of risk modifying drugs such as inhibitors of the renin-angiotensin system. Likewise, the relationship between increasing blood pressure and troponin reinforces the importance of optimisation of blood pressure-lowering therapies. Finally, troponin-guided care may have particular relevance when considering the appropriate use of novel therapeutic agents such as inhibitors of proprotein convertase subtilisin–kexin type 9^26^ and the anti-inflammatory drug canakinumab.^27^ Accurate risk stratification may enable more rational use of these drugs which are expensive and associated with potential adverse effects that may otherwise limit broad clinical application.

We recognise that our study has some limitations. The biomarker used for the index diagnosis of myocardial infarction varied between study centres and included the troponin T and troponin I assays available during the period of study recruitment. This precludes a direct comparison of the relationship between the peak high-sensitivity troponin concentration and those measured using the high-sensitivity assay during follow-up. However, peak creatine kinase concentrations were available in 91% of patients and have previously been demonstrated to correlate with size of infarct and clinical outcomes.^28 29^ Using peak creatine kinase, we identified only a weak relationship with convalescent troponin values and no correlation with cardiovascular death within the fully adjusted model. This may reflect changes in contemporary management of the patients enrolled in the CDCS with high rates of coronary revascularisation and use of secondary prevention medications. Finally, although analytical variation is modest, even at low concentrations, some variation in reported troponin concentrations is possible when testing is performed across different analysers and using different reagent batches. Such variation can be kept to a minimum through the careful application of quality control measures as recommended by current guidelines.^30^

We conclude that the measurement of high-sensitivity cardiac troponin in the convalescent phase following acute coronary syndrome identifies an important group of patients at high risk of recurrent cardiac events and death. Recognition of this increased risk may allow better targeting of secondary prevention therapies and could improve the clinical impact of future therapeutic trials in patients with acute coronary syndrome.

**Key messages**

**What is already known on this subject?**

- In the diagnostic assessment of acute coronary syndromes, serial measurement of plasma cardiac troponin concentrations is recommended by European and North American guidelines. In this context, the detection of troponin concentrations above the 99th centile upper reference limit is associated with increased short-term risk and may help inform early management decisions regarding the need for hospital admission and inpatient coronary angiography. However, the prognostic value and optimal timing for measurement of convalescent troponin following hospital discharge remain unknown.

**What might this study add?**

- We investigated the long-term prognostic significance of serial convalescent high-sensitivity troponin I concentrations following acute coronary syndrome in a large prospective multicentre observational cohort study with detailed clinical, biochemical and echocardiographic characterisation. We identified a strong association between 4-month troponin concentrations and cardiovascular death over 5 years that was independent of the extent of myocardial injury during the index event and all other established risk factors. Repeated measurement of troponin at 12 months provided additional prognostic information.

**How might this impact on clinical practice?**

- Low plasma cardiac troponin I concentrations measured 4 months following an index hospitalisation with acute coronary syndrome identified nearly half of all patients as being at very low risk of long-term cardiovascular death. In contrast, 1 in 10 patients have persistent myocardial injury and are at very high risk. These individuals clearly warrant close follow-up, with concerted efforts made to optimise their medical care. For the remaining intermediate-risk patients, repeated measurement of troponin concentrations at 12 months enables prognostic reclassification into either low-risk or high-risk groups.
