# Supplementary material for: Convalescent troponin and cardiovascular death following acute coronary syndrome
Source: Heart. 2019 Jul 23;105(22):1717–24. doi: 10.1136/heartjnl-2019-315084 (PMC6855795; doi:10.1136/heartjnl-2019-315084)
Supplement: Supplementary data [file heartjnl-2019-315084supp001.docx]

SUPPLEMENTARY APPENDIX

**Convalescent Troponin and Cardiovascular Death following Acute Coronary Syndrome**

Philip D. Adamson MD,^1,3^ David A. McAllister MD,^2^ Anna Pilbrow PhD,^3^ John W. Pickering PhD,^3^ Katrina K. Poppe PhD,^4^ Anoop S.V. Shah MD,^1^ Gillian A. Whalley PhD,^5^ Chris J. Ellis, BM,^4^ Nicholas L. Mills MD,^1^ David E. Newby MD,^1^ Chris J Pemberton PhD,^3^ Richard Troughton MD,^3^ Robert N. Doughty MD,^4,6^ A. Mark Richards MD,^3,7^

### Affiliations

^1^BHF Centre for Cardiovascular Science, University of Edinburgh, Edinburgh, United Kingdom

^2^Institute of Health and Wellbeing, University of Glasgow, Glasgow, United Kingdom

^3^Christchurch Heart Institute, University of Otago, Christchurch, New Zealand

^4^Faculty of Medical and Health Sciences, University of Auckland, New Zealand

^5^Dunedin School of Medicine, University of Otago, New Zealand

^6^Greenlane Cardiovascular Service, Auckland City Hospital, Auckland, New Zealand

^7^Cardiovascular Research Institute, National University of Singapore, Singapore

### Address for correspondence:

Dr Philip D. Adamson

BHF Centre for Cardiovascular Science

Chancellor’s Building

University of Edinburgh

49 Little France Crescent

Edinburgh

EH16 4SB

United Kingdom

Email: [philip.adamson@ed.ac.uk](mailto:philip.adamson@ed.ac.uk)

**Supplementary Figures:** 6

**Supplementary Tables:** 4

## Table of Contents

**Page Number**

1. **Expanded Methods**
2. **Supplementary Figures**

Figure S1

Figure S2

Figure S3

Figure S4

Figure S5

Figure S6

1. **Supplementary Tables**

Table S1

Table S2

Table S3

Table S4

**3**

**6**

**7**

**8**

**9**

**10**

**11**

**12**

**13**

**14**

**15**

**14-15**

## Expanded Methods

### Inclusion criteria

Inclusion required hospitalization with an acute coronary syndrome diagnosed in patients presenting within 72 hours after the onset of ischemic discomfort. In the absence of biochemical evidence of myocardial necrosis, a diagnosis of unstable angina required the presence of ischemic changes on the electrocardiogram (ST-segment depression or elevation of at least 0.5 mm, T-wave inversion of at least 3 mm in at least 3 leads, or left bundle branch block), or an established history of coronary artery disease, or age ≥65 years in patients with diabetes or established vascular disease.(1)

### Endpoint identification

Deaths were adjudicated for cause according to the American College of Cardiology Task Force on Clinical Data Standards.(2) Specifically, cardiovascular death indicates cause of death was sudden cardiac death, myocardial infarction, unstable angina, or other coronary artery disease; vascular death (e.g., stroke, arterial embolism, pulmonary embolism, ruptured aortic aneurysm, or dissection); congestive heart failure; or cardiac arrhythmia. Non-fatal events were determined from ICD-codes at time of hospital discharge obtained via New Zealand’s National Health Information Service. The following ICD-10 codes were used to determine the secondary endpoints.

Cardiovascular events: I20-I25 (all sub-codes)

Myocardial infarction: I21-I22 (all sub-codes)

Heart failure hospitalisation: I50.0, I50.1, I50.9

Ischaemic stroke: I63-I66 (all sub-codes)

Haemorrhagic stroke: I60-I62 (all sub-codes)

### Statistical analysis

Patient characteristics were reported by groups determined from the 4-month troponin concentration rounded to the nearest integer value, in keeping with usual clinical practice. Groups 1 and 2 comprised those below and above the median troponin concentration excluding those in group 3 with troponin concentrations above the sex-specific 99^th^ centiles. For modelling purposes, variables with a skewed distribution (including troponin concentrations) were log-transformed. Using troponin as a continuous marker, outcomes were analyzed with Cox regression. Results are reported as hazard ratios per doubling of troponin concentration after testing the assumption of proportional hazards in an unadjusted model and following both adjustment for the Global Registry of Acute Coronary Events (GRACE) risk score at time of discharge following index hospitalisation and an extended risk model incorporating additional clinical, biochemical and echocardiographic factors during follow-up to 4 months.(3) Survival curves were constructed according to troponin groups.

Receiver operating characteristic curves were created and discrimination of peak creatine kinase during index hospital admission and 4-month troponin I concentrations for the endpoint of cardiovascular death at 5 years was compared using the Delong method.(4)

In determining the relationship between troponin concentrations and treatment we defined optimal medical therapy at discharge as per current clinical guidelines.(5) This required all patients to be prescribed aspirin, clopidogrel and a statin. In addition, prescription of a beta-blocker was required if the left ventricular ejection fraction (LVEF) was ≤40%. An angiotensin converting enzyme inhibitor or angiotensin receptor antagonist was also required if the LVEF was ≤40%, or the patient had a diagnosis of hypertension or diabetes mellitus.

Clinical determinants of cardiac troponin concentrations were determined from linear mixed models. The slope (time since initial presentation) and intercept were included as random effects. Time varying covariates such as blood pressure and left-ventricular ejection fraction were centred within-person as per Curran and Bauer.(6) In a sensitivity analysis, we repeated the modelling with time as a categorical variable, using an unstructured correlation matrix for the within-person errors.

The negative predictive values for the primary outcome were established across a range of troponin concentrations starting at 2 ng/L. European guidelines on cardiovascular disease prevention specify a 5% risk of cardiovascular death over 10 years as a threshold identifying individuals at increased risk, warranting therapeutic intervention.(7) Assuming a constant linear event rate, having excluded patients with an event in the first four months, this would correspond to a negative predictive value at 5 years of 97.5%. We therefore evaluated this threshold as an acceptable indicator of low risk. As the negative predictive value was expected to be >95%, confidence intervals were estimated by sampling from a Beta distribution (as the conjugate prior to binomial likelihood, with a Jeffrey’s prior) as proposed previously.(8)

In a landmark analysis following the 12-month visit, the additional prognostic information provided by serial changes in troponin concentration was determined using a Cox proportional hazards model that included (log-transformed) troponin concentration at 12 months adjusted for (log-transformed) troponin concentration at 4 months. To improve interpretability we presented this result both in tabular and diagrammatic forms (Supplementary Table S3 and Figure 4). In the table we have presented the data by simulating a range of troponin concentrations at both time points and using the results of the Cox model to predict the estimated hazard ratio for each combination. In the Figure we have adopted a similar approach with simulated troponin concentrations that cover the majority of the observed range within the CDCS population. Percent change in troponin concentration was determined as: $\frac{12 month troponin - 4 month troponin}{4-month troponin}$. Consequently, positive change values reflect a higher troponin concentration at 12 months compared with 4 months and *vice versa*. Patients were included in this analysis if they had troponin results available from all 3 study visits and had not experienced a recurrent myocardial infarction prior to the 12-month visit (n=1,513).

Data are presented as mean (± standard deviation [SD]) or median (interquartile range [IQR]) with 95% confidence intervals with two-tailed p values reported. Statistical analysis was performed using R v3.4.3 (R Foundation for Statistical Computing, Vienna, Austria).

## Supplementary Figures

**
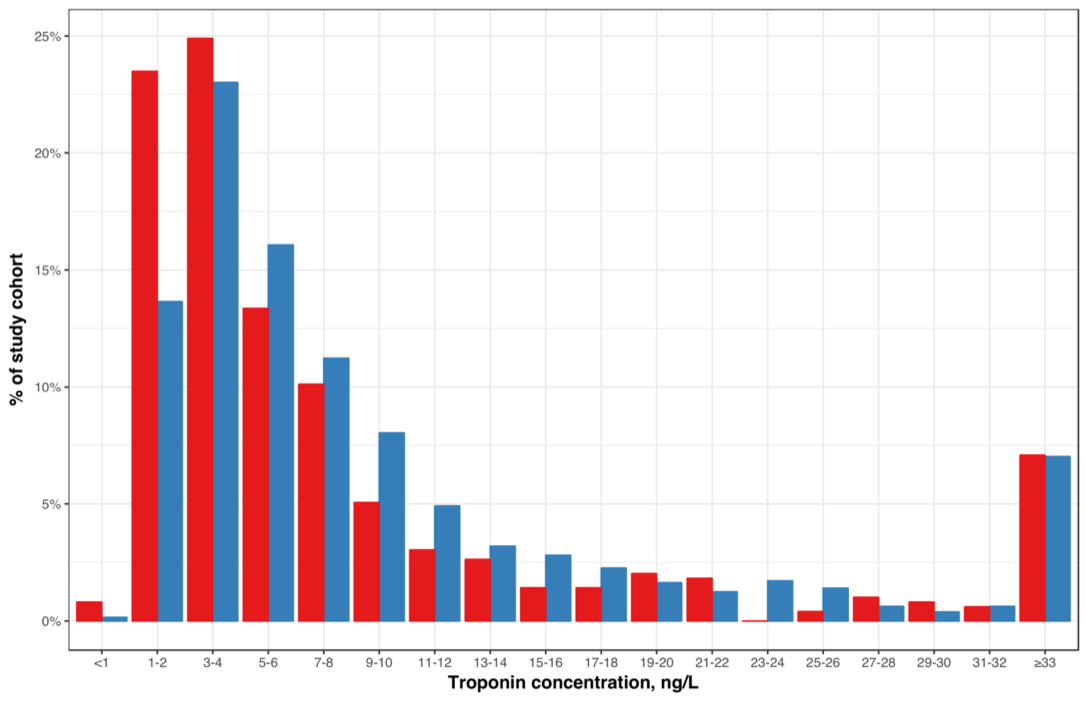
**

**Figure S1. Distribution of convalescent troponin concentrations at 4 months for women (red) and men (blue).**

**
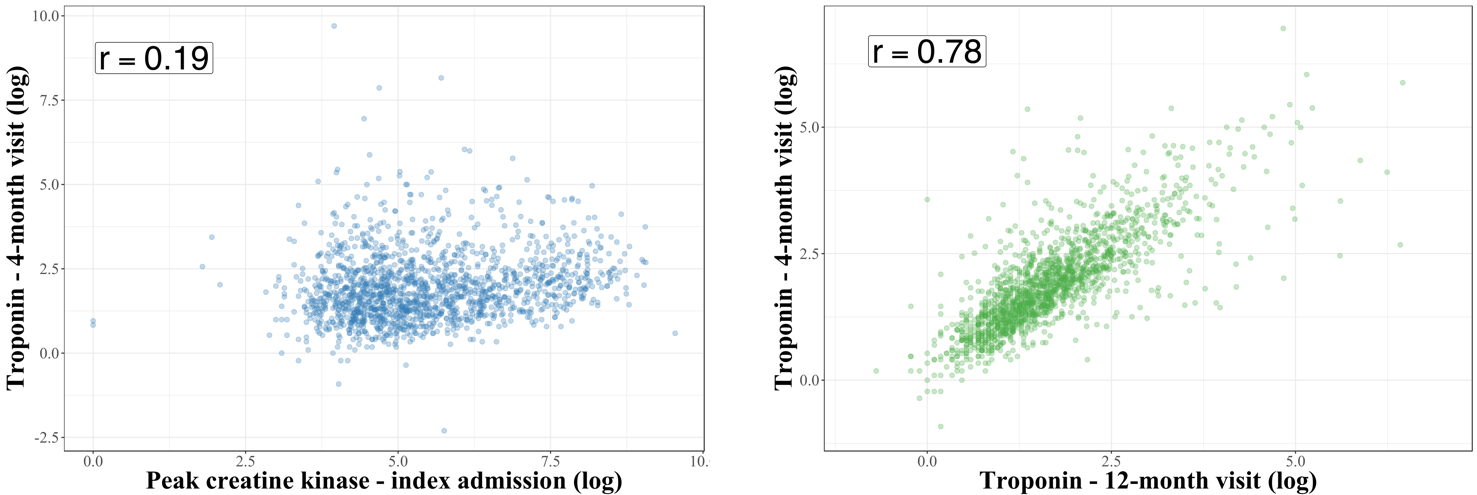
**

**Figure S3. Biomarker correlation from index admission to 12 months**

**
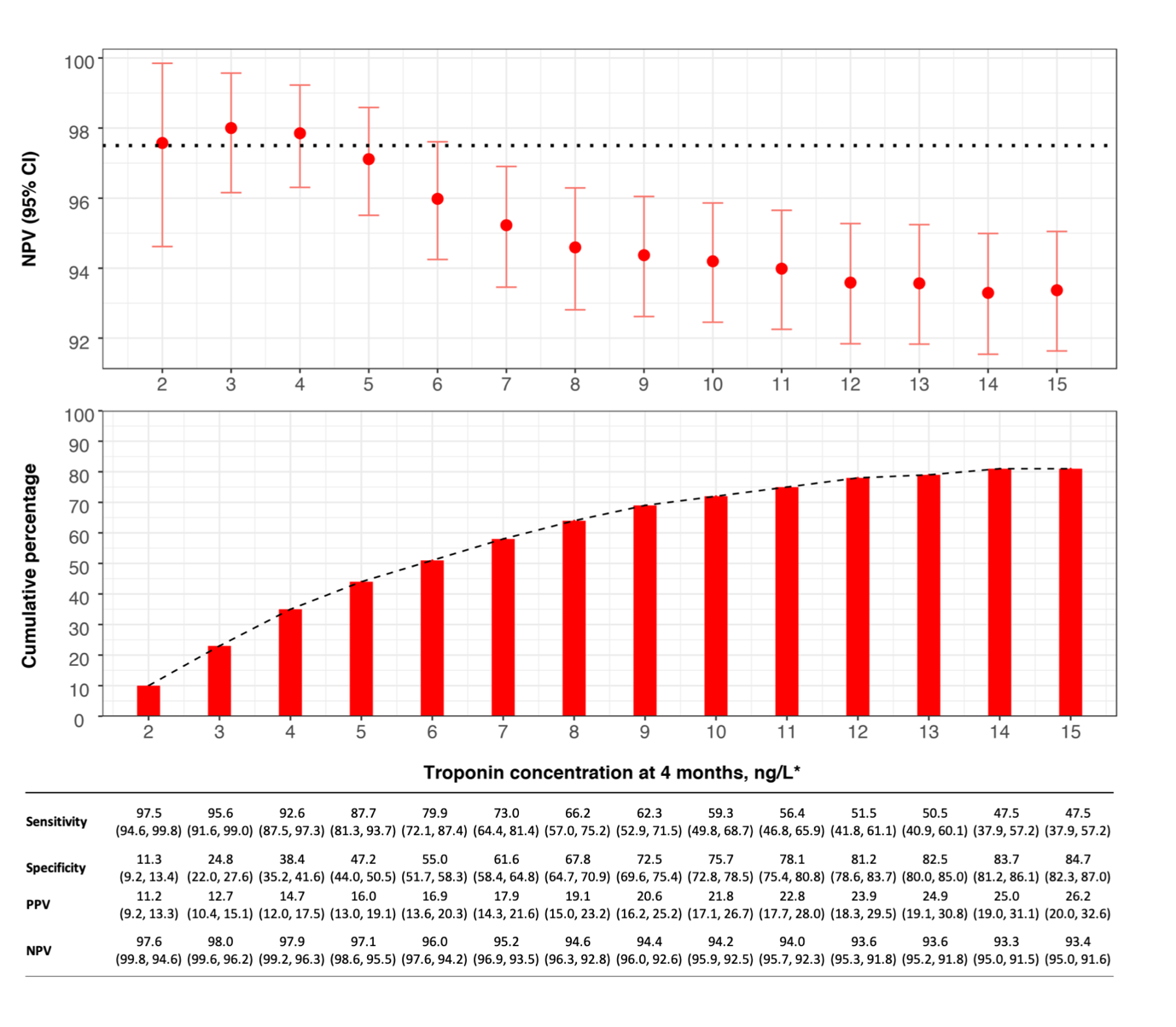
**

**Figure S3. Cardiac troponin I concentration at 4 months and 5-year risk of cardiovascular death.**

*(A) Negative predictive value across the range of troponin I concentrations at 4 months for the primary outcome of cardiovascular death at 5 years. (B) Cumulative proportion of study patients with troponin concentrations below each threshold. (C) Table prognostic metrics across the troponin concentrations (values in parentheses reflect 95% confidence intervals unless otherwise stated).*

*For descriptive purposes, troponin concentrations have been rounded to nearest integer value. Therefore a troponin concentration of 5ng/L includes all patients <5·5ng/L.

CI, confidence interval; NPV, negative predictive value; PPV, positive predictive value.


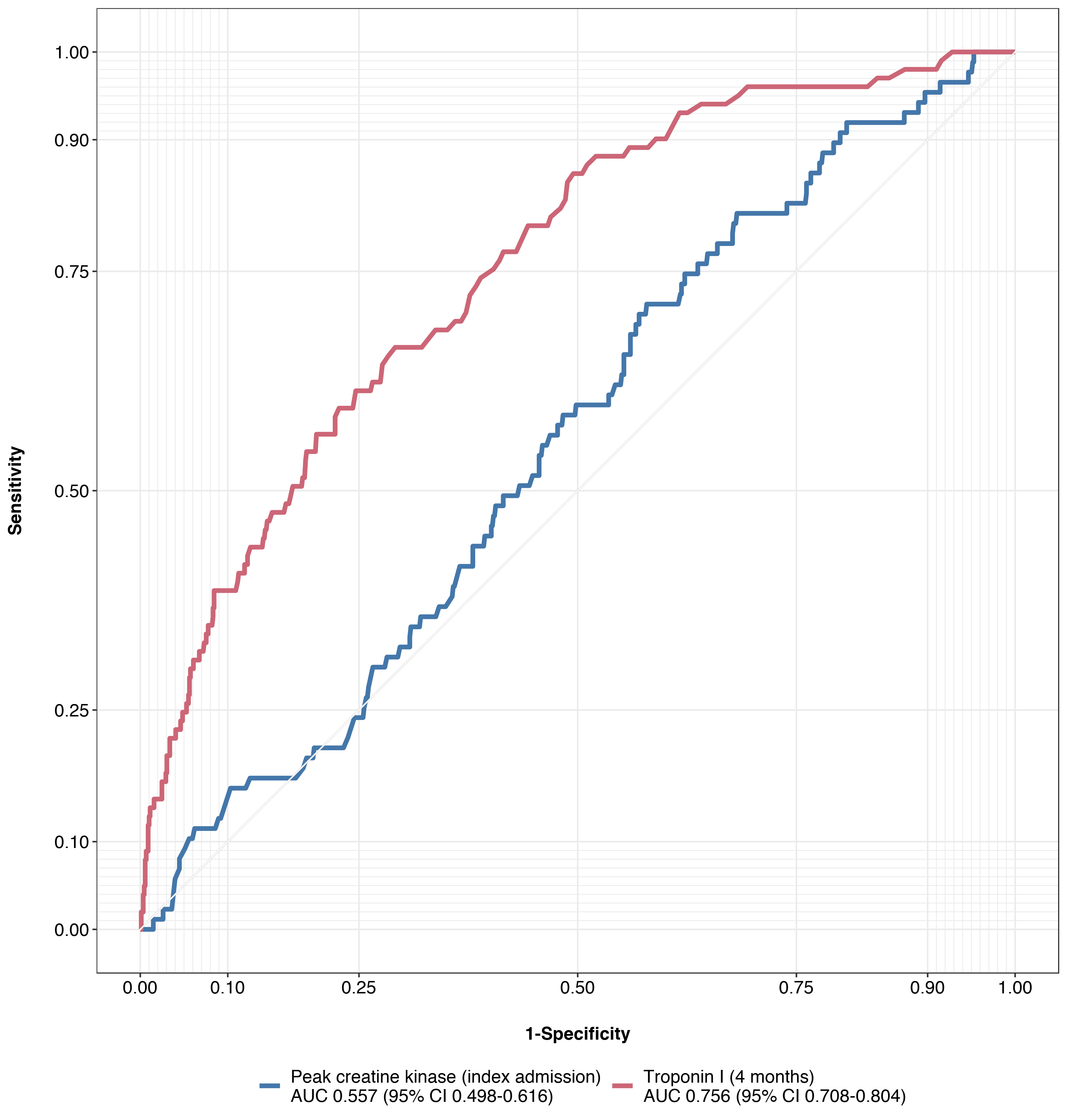


**Figure S4. Receiver operating characteristic curve for prediction of 5-year risk of cardiovascular death.**

*Comparison of convalescent troponin I measured at 4 months (red) with peak creatine kinase measured to determine the extent of myocardial injury during the index admission (blue) demonstrates greater discrimination of risk from the former (p<0·0001).*

AUC, area under curve; CI, confidence interval

**
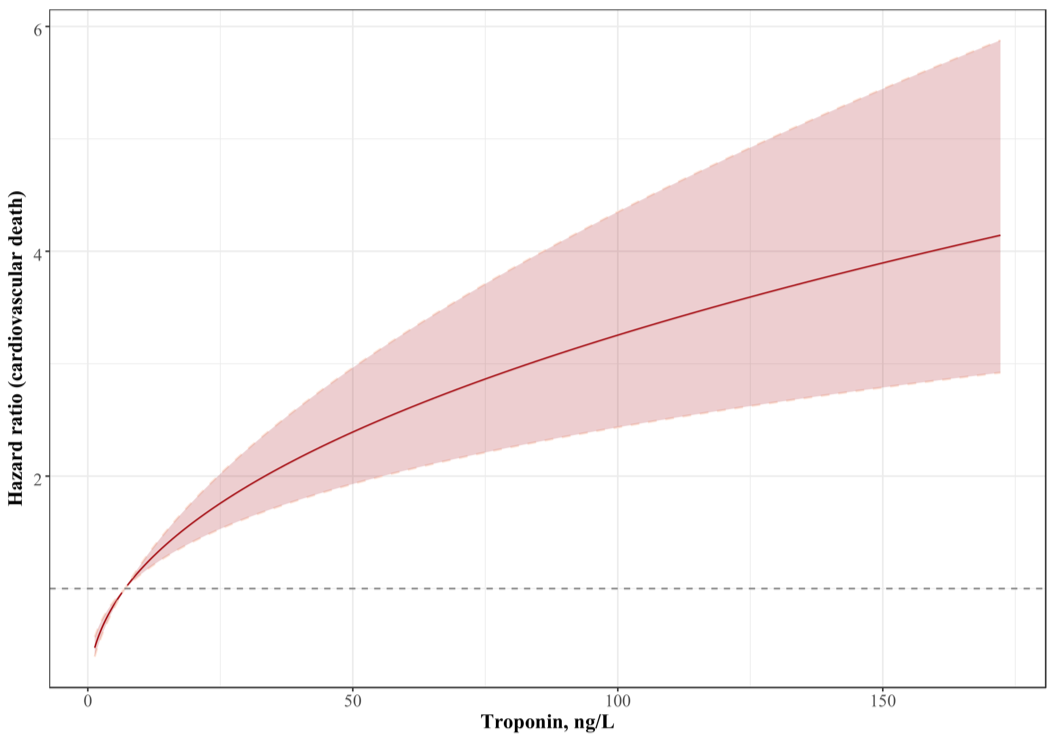
**

**Figure S5. Hazard ratio for cardiovascular death by troponin concentration at 4 months following acute coronary syndrome adjusted for GRACE risk.**

*High-sensitivity cardiac troponin I concentrations measured 4 months following acute coronary syndrome were log-transformed prior to analysis, and the hazard ratio for cardiovascular death over follow up of 5·0 years compares those above and below the median troponin concentration of 6·1 ng/L.*

##
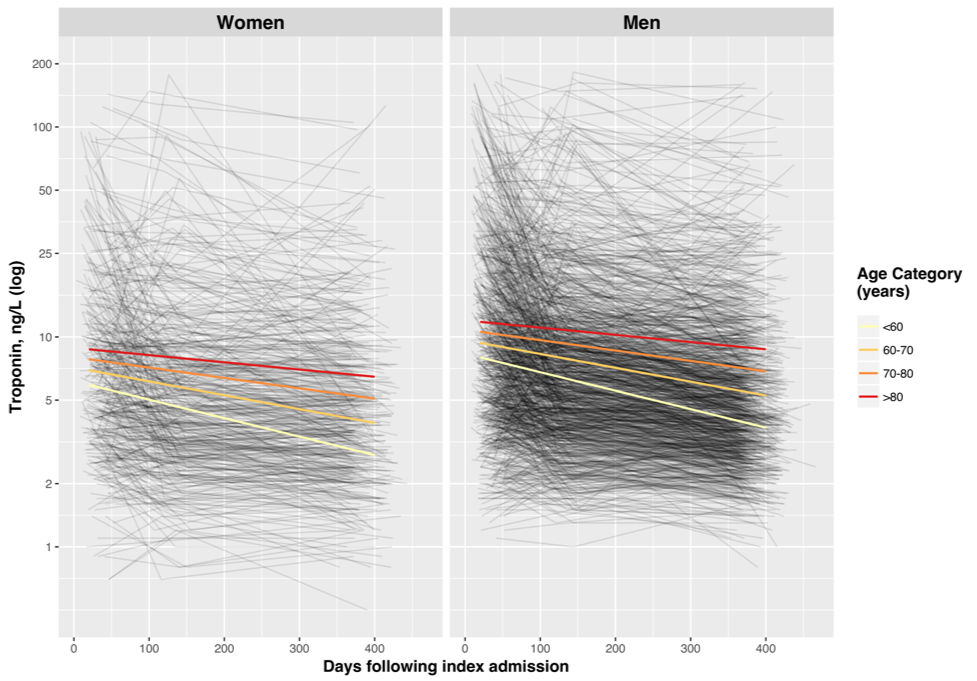


**Figure S6. Association between age and change in troponin concentration over time.**

*Individual patients are presented as grey lines with results for women (left panel) and men (right panel). The coloured lines depict the results of the linear mixed model demonstrating overall higher troponin concentrations at baseline and slower rate of decline in patients >80 years (red) compared with <60 years (yellow).*

## Supplementary Tables

**Table S1. Study-related procedures.**

|  | **1-month** | **4 months** | **12 months** | **24 months** | **36 months** |
| --- | --- | --- | --- | --- | --- |
| Check eligibility criteria | X |  |  |  |  |
| Obtain informed consent | X |  |  |  |  |
| Physical exam | X | X | X |  |  |
| Medical history  Family medical history  Admission details | X  X  X |  |  |  |  |
| Medications on admission | X |  |  |  |  |
| Medications at discharge | X |  |  |  |  |
| Current medications | X | X | X | X | X |
| Adverse Events | X | X | X | X | X |
| ECG | X | X | X |  |  |
| Echocardiograph | X | X | X |  |  |
| Neurohormonal blood samples | X | X | X |  |  |
| DNA blood sample | X |  |  |  |  |
| Questionnaire | X |  |  | X | X |
| NYHA, CCS Scores | X | X | X | X | X |

ECG, electrocardiogram; NYHA, New York Heart Association; CCS, Canadian Cardiovascular Society.

**Table S2. Multivariable Cox proportional hazards models for cardiovascular death**

|  | **Model 1 HR**  (95% CI) | **Model 2 HR**  (95% CI) | **Model 3 HR^¶^**  (95% CI) |
| --- | --- | --- | --- |
| **Troponin I at 4 months*, ng/L** | 1·50 ( 1·42 – 1·59 )§ | 1·38 ( 1·22 – 1·56 )§ | 1·35 ( 1·15 – 1·58 )§ |
| **Peak creatine kinase during index admission*, U/L** | – | 0·93 ( 0·80 – 1·08 ) | 0·87 ( 0·73 – 1·04 ) |
| **Age, years** | – | 1·07 ( 1·05 – 1·10 ) | 1·07 ( 1·04 – 1·10 ) |
| **Male** | – | 0·99 ( 0·58 – 1·67 ) | 0·82 ( 0·44 – 1·53 ) |
| **Creatinine*,  µmol/L** | – | 2·24 ( 1·36 – 3·70 ) | 1·57 ( 0·86 – 2·87 ) |
| **Heart failure during index admission** | – | 1·49 ( 0·94 – 2·37 ) | 1·28 ( 0·72 – 2·25 ) |
| **Index diagnosis†** | – | – | – |
| NSTEMI | – | 1·22 ( 0·67 – 2·23 ) | 2·25 ( 1·04 – 4·87 ) |
| STEMI | – | 1·38 ( 0·60 – 3·17 ) | 3·85 ( 1·36 – 10·92 ) |
| **Coronary disease burden**‡ | – | – | – |
| Single vessel disease | – | 0·69 ( 0·22 – 2·14 ) | 0·98 ( 0·22 – 4·26 ) |
| Multi-vessel disease | – | 1·17 ( 0·45 – 3·04 ) | 1·71 ( 0·48 – 6·14 ) |
| **Percutaneous coronary intervention not performed during index admission** | – | 1·96 ( 1·21 – 3·17 ) | 1·94 ( 1·10 – 3·43 ) |
| **Left ventricular ejection fraction at 4 months*, %** | – | – | 0·42 ( 0·22 – 0·78 ) |
| **Medical history prior to index admission** | – | – | – |
| Hypertension or hypercholesterolemia | – | – | 2·02 ( 0·90 – 4·50 ) |
| Diabetes mellitus | – | – | 1·03 ( 0·55 – 1·93 ) |
| Congestive heart failure or myocardial infarction | – | – | 1·71 ( 0·97 – 3·00 ) |
| Smoking status | – | – | – |
| Current or former smoker | – | – | 1·02 ( 0·58 – 1·80 ) |

§p=0·0001

**^¶^**Since model 3 contained a small number of events per variable, in sensitivity analyses we repeated the modelling for every possible combination of from 5 to 14 variables selected from the 14 variables shown. The association for troponin at 4-months remained robust, ranging from 1·27 (95% CI 1·17 to 1·38) for the weakest association and 1·64 (95% CI 1·48 to 1·82) for the strongest association.

*Variables log-transformed and coefficients should be interpreted per doubling.

†Hazard ratio compared to index diagnosis of unstable angina.

‡Coronary artery disease burden determined as number of major epicardial vessels with >50% diameter stenosis reported from diagnostic coronary angiogram during index admission. Hazard ratio compared to no obstructive disease identified on coronary angiogram.

HR, hazard ratio; CI, confidence interval; NSTEMI, non-ST elevation myocardial infarction; STEMI, ST elevation myocardial infarction.

**Table S3. Cox proportional hazard ratios for cardiovascular death according to troponin at each study visit**

|  |  | **Troponin concentration at 12 months, hazard ratio (95% CI)** | | | | |  |
| --- | --- | --- | --- | --- | --- | --- | --- |
| **Troponin concentration at 4 months** | | **2 ng/L** | **4 ng/L** | **8 ng/L** | **16 ng/L** | **32 ng/L** | |
|  | **2 ng/L** | – | 2·15 (1·61 – 2·87) | 4·61 (3·30 – 6·42) | 9·88 (6·19 – 15·78) | 21·21 (11·19 – 40·21) | |
|  | **4 ng/L** | 1·30 (1·06 – 1·59) | 2·62 (2·39 – 2·88) | 5·27 (4·37 – 6·36) | 10·60 (7·49 – 15·01) | 21·32 (12·75 – 35·67) | |
|  | **8 ng/L** | 1·70 (1·32 – 2·18) | 3·20 (2·80 – 3·65) | 6·03 (5·24 – 6·94) | 11·37 (8·74 – 14·79) | 21·44 (14·27 – 32·20) | |
|  | **16 ng/L** | 2·21 (1·40 – 3·48) | 3·90 (2·80 – 5·45) | 6·90 (5·36 – 8·88) | 12·19 (9·47 – 15·70) | 21·55 (15·44 – 30·09) | |
|  | **32 ng/L** | 2·88 (1·45 – 5·72) | 4·77 (2·78 – 8·16) | 7·89 (5·24 – 11·88) | 13·08 (9·46 – 18·08) | 21·67 (15·73 – 29·84) | |

Hazard ratios determined from a Cox proportional hazards model without additional adjustment for clinical covariates.

Using troponin concentration as a continuous variable, the hazard ratio per doubling of troponin was 1·39 (95% CI 1·04 to 1·86) at 4 months (for patients with a 12-month troponin concentration equal to the mean value) and 2·29 (95% CI 1·80 to 2·92) at 12 months (for patients with a 4-month troponin concentration equal to the mean value). Both associations were attenuated when the other measure was increased above the mean (p-interaction=0·0200).

CI, confidence interval.

**Table S4A. Between individual and within individual variation in troponin according to clinical features.**

|  | Model, coefficient (standard error) | | | | |
| --- | --- | --- | --- | --- | --- |
| Term | **1*** | **2†** | **3** | **4** | **5** |
| (Intercept) | 1·94 (0·02) | 1·93 (0·02) | 1·61 (0·04) | 1·61 (0·04) | 1·62 (0·04) |
| Age |  |  | 0·27 (0·02) | 0·28 (0·02) | 0·22 (0·02) |
| Sex |  |  | 0·12 (0·02) | 0·13 (0·02) | 0·09 (0·02) |
| Index diagnosis‡ |  |  |  |  |  |
| NSTEMI |  |  | 0·32 (0·05) | 0·32 (0·05) | 0·32 (0·05) |
| STEMI |  |  | 0·71 (0·06) | 0·71 (0·06) | 0·63 (0·06) |
| Between patient change |  |  |  |  |  |
| Left ventricular ejection fraction |  |  |  |  | -0·31 (0·02) |
| Systolic blood pressure |  |  |  |  | 0·07 (0·03) |
| Heart rate |  |  |  | 0·07 (0·03) | 0·02 (0·03) |
| Within patient change |  |  |  |  |  |
| Left ventricular ejection fraction |  |  |  |  | -0·04 (0·02) |
| Systolic blood pressure |  |  |  |  | -0·04 (0·02) |
| Heart rate |  |  |  | 0·03 (0·01) | 0·03 (0·01) |
| Time |  | -0·53 (0·02) | -0·17 (0·04) | -0·17 (0·04) | -0·18 (0·04) |
| Time:Age interaction |  |  | 0·09 (0·02) | 0·08 (0·02) | 0·09 (0·02) |
| Time:STEMI diagnosis interaction |  |  | -0·30 (0·05) | -0·30 (0·05) | -0·29 (0·05) |
| Time:NSTEMI diagnosis interaction |  |  | -0·92 (0·06) | -0·91 (0·06) | -0·87 (0·06) |
| Standard deviations for variation |  |  |  |  |  |
| Between participant intercept | 0·80 | 0·82 | 0·75 | 0·75 | 0·72 |
| Between participant slope |  | 0·42 | 0·23 | 0·26 | 0·23 |
| Within participant residual | 0·64 | 0·53 | 0·53 | 0·53 | 0·51 |
|  |  |  |  |  |  |

*Unconditional model (random intercept only); †Unconditional growth model (random intercept and slope)

‡Hazard ratio compared to index diagnosis of unstable angina.

*This table shows the coefficients and estimated standard deviations for linear mixed models of log-troponin on covariates. The intercept and slope are modelled as random effects, with the remaining variables being modelled as fixed effects. As well as the standard deviation for the between-person variation in the intercept and slope, the table shows the standard deviation for the within-person residual error. All models are fitted using restricted maximum likelihood. All results have been presented on the original scale, but can be exponentiated to obtain the relative difference scale as reported in the manuscript (e.g. coefficient = 0·2 corresponds to a 1·22-fold difference – see* ***Table S4B****). Time was standardised for the model fitting to have a standard deviation of 1, but the time (and time-covariate interaction) coefficients have subsequently been transformed so that these can be interpreted as the difference in troponin per-year.*

NSTEMI, non ST-segment elevation myocardial infarction; STEMI, ST-segment elevation myocardial infarction.

**Table S4B. Between individual and within individual variation in troponin according to clinical features (exponentiated).**

|  | Model, relative difference (95% CI) | | | | |
| --- | --- | --- | --- | --- | --- |
| Term | **1*** | **2†** | **3** | **4** | **5** |
| Age |  |  | 1·31 (1·26 – 1·36) | 1·32 (1·27 – 1·38) | 1·25 (1·20 – 1·30) |
| Sex |  |  | 1·13 (1·08 – 1·17) | 1·14 (1·10 – 1·18) | 1·09 (1·05 – 1·14) |
| Index diagnosis‡ |  |  |  |  |  |
| NSTEMI |  |  | 1·38 (1·25 – 1·52) | 1·38 (1·25 – 1·52) | 1·38 (1·25 – 1·52) |
| STEMI |  |  | 2·03 (1·81 – 2·29) | 2·03 (1·81 – 2·29) | 1·88 (1·67 – 2·11) |
| Between patient change |  |  |  |  |  |
| Left ventricular ejection fraction |  |  |  |  | 0·73 (0·71 – 0·76) |
| Systolic blood pressure |  |  |  |  | 1·07 (1·01 – 1·14) |
| Heart rate |  |  |  | 1·07 (1·01 – 1·14) | 1·02 (0·96 – 1·08) |
| Within patient change |  |  |  |  |  |
| Left ventricular ejection fraction |  |  |  |  | 0·96 (0·92 – 1·00) |
| Systolic blood pressure |  |  |  |  | 0·96 (0·92 – 1·00) |
| Heart rate |  |  |  | 1·03 (1·01 – 1·05) | 1·03 (1·01 – 1·05) |
| Time |  | 0·59 (0·57 – 0·61) | 0·84 (0·78 – 0·91) | 0·84 (0·78 – 0·91) | 0·84 (0·77 – 0·90) |
| Time:Age interaction |  |  | 1·09 (1·05 – 1·14) | 1·08 (1·04 – 1·13) | 1·09 (1·05 – 1·14) |
| Time:STEMI diagnosis interaction |  |  | 0·74 (0·67 – 0·82) | 0·74 (0·67 – 0·82) | 0·75 (0·68 – 0·83) |
| Time:NSTEMI diagnosis interaction |  |  | 0·40 (0·35 – 0·45) | 0·40 (0·36 – 0·45) | 0·42 (0·37 – 0·47) |

NSTEMI, non ST-segment elevation myocardial infarction; STEMI, ST-segment elevation myocardial infarction.

## Supplementary References

1. de Lemos JA, Morrow DA, Bentley JH, Omland T, Sabatine MS, McCabe CH, et al. The prognostic value of B-type natriuretic peptide in patients with acute coronary syndromes. The New England journal of medicine. 2001;345(14):1014-21.

2. Cannon CP, Battler A, Brindis RG, Cox JL, Ellis SG, Every NR, et al. American College of Cardiology key data elements and definitions for measuring the clinical management and outcomes of patients with acute coronary syndromes. A report of the American College of Cardiology Task Force on Clinical Data Standards (Acute Coronary Syndromes Writing Committee). Journal of the American College of Cardiology. 2001;38(7):2114-30.

3. Eagle KA, Lim MJ, Dabbous OH, Pieper KS, Goldberg RJ, Van de Werf F, et al. A validated prediction model for all forms of acute coronary syndrome: estimating the risk of 6-month postdischarge death in an international registry. JAMA : the journal of the American Medical Association. 2004;291(22):2727-33.

4. DeLong ER, DeLong DM, Clarke-Pearson DL. Comparing the areas under two or more correlated receiver operating characteristic curves: a nonparametric approach. Biometrics. 1988;44(3):837-45.

5. Roffi M, Patrono C, Collet JP, Mueller C, Valgimigli M, Andreotti F, et al. 2015 ESC Guidelines for the management of acute coronary syndromes in patients presenting without persistent ST-segment elevation: Task Force for the Management of Acute Coronary Syndromes in Patients Presenting without Persistent ST-Segment Elevation of the European Society of Cardiology (ESC). European heart journal. 2016;37(3):267-315.

6. Curran PJ, Bauer DJ. The disaggregation of within-person and between-person effects in longitudinal models of change. Annual review of psychology. 2011;62:583-619.

7. Perk J, De Backer G, Gohlke H, Graham I, Reiner Z, Verschuren M, et al. European guidelines on cardiovascular disease prevention in clinical practice (version 2012). The Fifth Joint Task Force of the European Society of Cardiology and Other Societies on Cardiovascular Disease Prevention in Clinical Practice (constituted by representatives of nine societies and by invited experts). European heart journal. 2012;33(13):1635-701.

8. Brown LD, Cai TT, DasGupta A. Interval estimation for a binomial proportion. Statistical Science. 2001;16(2):101-17.
